# Coronavirus Awareness and Mental Health: Clinical Symptoms and Attitudes Toward Seeking Professional Psychological Help

**DOI:** 10.3389/fpsyg.2021.549644

**Published:** 2021-04-22

**Authors:** Miguel Landa-Blanco, Ana Landa-Blanco, Claudio J. Mejía-Suazo, Carlos A. Martínez-Martínez

**Affiliations:** ^1^Clinical Psychology, Faculty of Social Sciences, School of Psychological Sciences, National Autonomous University of Honduras, Tegucigalpa, Honduras; ^2^Social and Economic Research, Faculty of Economy and Management, National Autonomous University of Honduras, Tegucigalpa, Honduras; ^3^Faculty of Sciences, School of Biology, National Autonomous University of Honduras, Tegucigalpa, Honduras; ^4^Faculty of Medical Sciences, National Autonomous University of Honduras, Tegucigalpa, Honduras

**Keywords:** pandemics, mental health, psychological help, health psychology, public health

## Abstract

The current study analyzed the relationship between Coronavirus (COVID-19) Awareness, mental health, and willingness to seek professional psychological help. This was made through a quantitative approach, using online questionnaires to collect data from 855 subjects. The questionnaires included the Brief Symptom Inventory (BSI-53) to measure mental health indicators, the Attitudes Toward Seeking Professional Psychological Help Scale–Short Form, and the Coronavirus Awareness Scale-10 (CAS-10). An Exploratory Factor Analysis suggests that three factors underlie the CAS-10: Coronavirus Concern, Exaggerated Perception, and Immunity Perception. Results indicate a significant positive correlation between Coronavirus Concern and both general anxiety and phobic anxiety symptoms. Immunity Perception is positively related to paranoid ideation and psychotic symptoms. A Mediation Analysis determined that Coronavirus Concern has a significant positive direct effect on Openness to Seeking Psychological Treatment (OSPT), while Exaggerated Perception and Immunity Perception scores have significant direct negative effects on the Value and Need in Seeking Treatment (VNST) scores. Indirectly, the relationship between Coronavirus Concern and OPST is significantly mediated by anxiety symptoms. Similar results were found for the VNST subscale. There is a negative significant effect of Immunity Perception over OSPT mediated by Paranoid Ideation. However, the overall model only achieved small *r*^2^ coefficients for the OSPT (0.060) and VNST (0.095) scores. Comparisons in Coronavirus Awareness between sex, age, and the presence of children and older adults at home were also made. These results are discussed regarding their practical implications for mental health providers and policymakers.

## Introduction

### Origins of COVID-19

Coronavirus is one of the most important pathogens that causes respiratory infections in humans. On December 2019, an outbreak of pneumonia of unknown cause was reported in the city of Wuhan, China. By January 2020, the pathogen was isolated from these patients and was identified as a novel Coronavirus (Severe Acute Respiratory Syndrome-Coronavirus 2). It was highly suspected that the outbreak started in a Huanan seafood market, a place where live animals such as bats, birds, snakes and frogs were sold (Shereen et al., 2020). This disease is highly contagious, initially from zoonotic transmission, and later from human to human, by coughing, sneezing or having close contact with an infected person’s respiratory droplets (Rothan and Byrareddy, 2020; Shereen et al., 2020).

The most common symptoms of COVID-19 illness are fever, cough, dyspnea, and myalgia. Populations at higher risk include older adults and people with underlying conditions like diabetes, hypertension, or coronary heart disease (Mesa Vieira et al., 2020). In such cases, health complications can quickly progress to Acute Respiratory Distress Syndrome (ARDS) or end-organ failure (Wu et al., 2020).

On the other hand, the evidence shows that only a small number of children that are COVID-19 positive develop a severe health condition. However, asymptomatic children could be playing a relevant part in the spread of the virus (Dervrim and Bayram, 2020). It’s important to reduce the increasing cases to avoid a higher fatality rate, especially for healthcare systems that are not prepared for this kind of pandemic, like in the Latin American region (Rodríguez-Morales et al., 2020).

### Prevalence of COVID-19

The World Health Organization (2020a) at the beginning of 2020 considered the outbreak of COVID-19 an international public health emergency. The virus kept spreading quickly in a great number of territories around the world, by March of 2020 it was cataloged as a pandemic. By May 27th, 2020, this number increased considerably, registering 5,491,678 confirmed cases and 349,190 deaths worldwide, with a great prevalence of cases in the American region (World Health Organization, 2020b). The spreading of Coronavirus in Latin American countries has presented an aggressive dynamic that suggests a difficult scenario for low-income nations (Caicedo-Ochoa et al., 2020). In the case of Honduras, by the date data for this research was collected (16th–23rd of March), the virus was just beginning to spread, with 30 confirmed cases and 0 deaths. By May 27th, 2020, these numbers increased alarmingly, reaching 4,401 confirmed cases and 188 fatalities (Honduras Health Secretary, 2020).

### Mental Health in the Context of COVID-19

According to the World Health Organization (2003), mental health is an important part of the human condition, along with the physical and social domains. Mental health concepts are related to subjective well-being, autonomy, the capability of identifying one’s potential, the ability to manage stress, work in a productive way and be capable of contributing to their social environment. Mental health is associated with a balance between the person and the environment. This is influenced by a series of biological, psychological, social, and cultural factors (Korkeila et al., 2003). Recent outbreaks such as SARS, Zika, MERS, and Ebola have shown that a health crisis is a stressful situation. These concerns may be related to the risk of acquiring the virus or passing it on to others, the presence of symptoms of other health conditions that could be confused with COVID-19, and physical and mental health deterioration in vulnerable populations. Other concerns are related to the uncertainty of the long-term consequences in the health, social and economic domains (Huremovié, 2019; Inter-Agency Standing Committee, 2020; Wang et al., 2020).

The constant fear of becoming infected or dying, as well as seeing other people die, are just one of the effects caused by outbreaks of epidemics and pandemics on mental health. People who become infected may be attacked and marginalized because they are perceived as “contaminated” (Huremovié, 2019; Wang et al., 2020). Likewise, there is a direct correlation between the growing crisis and the negative impact on the economy, health and educational systems (Sim et al., 2010; Van Bortel et al., 2016). This vulnerability can also be related to mental health issues to specific populations like older adults (El Hayek et al., 2020).

One of the most relevant public health measures to reduce the number of people infected with COVID-19 has been social distancing and quarantine (Wilder-Smith et al., 2020). Quarantine has shown to have negative psychological effects on people with and without pre-existing mental health problems. People under quarantine may experience symptoms related to anxiety, depression, and post-traumatic stress symptoms (Brooks et al., 2020). In addition, media can influence the public by doing “agenda-setting,” which occurs when a problem receives massive coverage, making it more important to the public (Rubin et al., 2010; Rubin and Wessly, 2020). People’s lack of knowledge about a disease leads to misinformation and the spread of rumors, which could lead to harmful effects on mental health (Fernández Poncela, 2012).

Although there are many instruments designed to screen psychological symptoms, the Brief Symptom Inventory-53 (BSI-53) has been widely used in different contexts. The BSI-53 measures symptoms related to Anxiety, Depression, Phobic Anxiety, Hostility, Interpersonal Sensitivity, Obsessive-Compulsive traits, Paranoid Ideation, Psychoticism, and Somatization (Derogatis, 1993). The following description provides a brief overview of these symptoms during the pandemic and confinement period:

• Anxiety: the contextual presence of different diseases or viruses can cause anxiety in the general population. Specific outbreaks (like Zika, Ebola, etc.) may detonate diverse patterns of health anxiety responses (Blakey and Abramowitz, 2017). Recent research made within the COVID-19 outbreak suggests a high prevalence of anxiety symptoms among the population (Rajkumar, 2020).
• Depression: has been described as one of the most prevalent symptoms among the general population during the current COVID-19 pandemic (Rajkumar, 2020).
• Phobic anxiety: recent studies have concluded that the fear of COVID-19 is related to variables such as perceived infectability and germ aversion (Kwasi Ahorsu et al., 2020).
• Hostility: during the SARS outbreak of 2003, health-care workers who were in quarantine reported high levels of anger, frustration, and annoyance (Brooks et al., 2020).
• Interpersonal sensitivity: is defined as a disproportionate awareness of other people’s conducts and emotions (Mushtaq et al., 2017). Limited research is available on the topic of interpersonal sensitivity in the context of COVID-19. However, there is evidence that suggests that such construct is an important factor when promoting social functioning in help-seeking individuals (Masillo et al., 2015).
• Obsessive-Compulsive symptoms: the COVID-19 outbreak may trigger such symptoms. This can be exacerbated by the biosecurity measures taken to prevent COVID-19, such as hand washing (quality and frequency), interaction with others with suspected exposure, excessive mediatic information, etcetera (Debanjan, 2020).

Paranoid Ideation: recent research suggests that subjects with a history of paranoid ideation may report high levels of fear related to COVID-19 transmission (Vinkers et al., 2020). A study in Indian population reported that a significant percentage of respondents showed health-related paranoia regarding COVID-19 (Roy et al., 2020).

• Psychoticism: a recent study concluded that some subjects who tested positive on COVID-19 presented stress-triggered psychotic symptoms. However, further research is yet needed on the subject to explore alternatives explications to this reaction (Ferrando et al., 2020).
• Somatization: is characterized by the presence of physical symptoms related to dysfunctional concerns. Recent evidence suggests that the current fear of COVID-19 infection may aggravate pre-existing conditions related to somatic symptom disorders (Colizzi et al., 2020).

However, the Latino population have a tendency to underestimate the relevance of mental health care (Liu et al., 2020; Torres et al., 2020). Latin American population tends to avoid seeking psychological help for fear of the diagnosis they may receive and its associated social stigma (Mascayano et al., 2015). Added to the above, are the religious and cultural beliefs in Latin America which could play an important role in the decision of not seeking psychological help (Caplan, 2019). Despite this cultural resistance, the School of Psychological Science of the National Autonomous University of Honduras, launched an online chat service to provide psychological help amidst the COVID-19 crisis. As of June 11, 2020, more than 711 persons had been attended through the application (School of Psychological Sciences, 2020). This data evidences the demand for mental health services among the Honduran population.

### COVID-19 Awareness

The present study takes into consideration Coronavirus Awareness in relation to mental health and attitudes toward seeking professional psychological help. Coronavirus Awareness is defined as the degree in which people are conscious of the meaning, implications, prevention strategies, and seriousness of the spreading of COVID-19. Recently, a study used Google Trends to analyze the search volume of queries regarding COVID-19 and related terms. The results indicate that people respond temporarily to local propaganda regarding the virus (indicating awareness), however, this attention spam had a short duration (Hu et al., 2020).

Research suggests that demographic variables, such as sex, may be related to Coronavirus Awareness. A study made within the United States of America (USA) context compared the proportion of female and male respondents who reported to be concerned about the COVID-19 situation. Results indicate that when compared to men, there is a higher proportion of women that claim to be concerned about their risk of exposure to COVID-19. Women are also more concerned about contagion risk in their families, loss of economic income and access to COVID-19 testing and treatment (Frederiksen et al., 2020).

Household configuration may also be related to Coronavirus Awareness. Men and women with children are more likely to report COVID-19 related concerns (risk of exposure and loss of economic income), when compared with people who do not have children (Frederiksen et al., 2020). In addition, older adults have a high COVID-19 physical vulnerability (Mesa Vieira et al., 2020) and are also exposed to the social, psychological and economic repercussions of the pandemic. Furthermore, quarantine measures have also promoted intergenerational cohesion, improving the bond older adults have with their own family members as well as with non-related younger people (Morrow-Howell et al., 2020). Age is another variable to consider when analyzing COVID-19 Awareness. Previous research in the United States concluded that the proportion of older adults (>60 years) reporting health-related concerns about the COVID-19 situation was higher than in younger adults (<60 years) (Hamel et al., 2020). However, no data is yet available for the Central American context.

### Purpose of the Study

**FIGURE 1:**
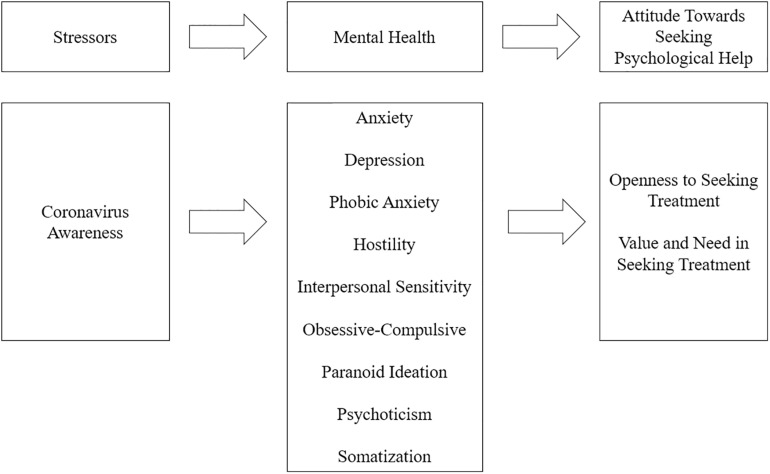
A proposed mediation model with Coronavirus Awareness as predictor, mental health symptoms as mediators and the attitude toward seeking psychological help as outcome.

Reflecting on what has been previously stated, the current study assumes the following premises: (a) the COVID-19 situation is considered a stressor (Wang et al., 2020), (b) stress is strongly related to the presence of mental health disorders (Wu et al., 2020), (c) subjective needs play an important role in help-seeking behaviors (Nagai, 2015); therefore, we propose to analyze a causal model based on these assumptions contextualized within the COVID-19 crisis (see Figure 1).

Consequently, the purpose of this study was to analyze the relationship between Coronavirus (COVID-19) Awareness with mental health indicators and the attitude toward seeking professional psychological help. Additional information regarding Coronavirus Awareness and demographic variables were also analyzed, such variables included: age, sex, presence of children and older adults at home. To our knowledge, there are no studies in Honduras or the Central American region that evaluate the impact of the COVID-19 situation in mental health.

## Materials and Methods

### Participants

#### Sampling Method

A total of 855 participants from Honduras answered an online survey that was spread through social media and by snow-ball sampling. This online method was selected given the country’s quarantine regulations. Each survey was accompanied by an online informed consent which stated the purpose of the study, a confidentiality clause and the main researcher’s contact information. The selection criteria for the participants included: (a) being 18 years or older, (b) currently residing in Honduras and, (c) agreeing with the informed consent statement; any violation of these criteria was considered a motive for exclusion.

#### Characteristics of the Participants

Of all the respondents, 307 (35.9%) were male and 548 (64.1%) female. The average age for male participants was of 27.619 years (*SD* = 10.284), while female mean age was 28.755 (*SD* = 10.878), however, this difference is not statistically significant, *t* (853) = -1.494, *p* = 0.135. On the other hand, 645 (75.4%) respondents were single, while the remaining 210 (24.6%) were married. Regarding household configuration, 444 (51.9%) respondents reported to live at home with children under 12 years old, while 327 (38.2%) lived at home with people 60 years or older.

### Measures

#### Brief Symptom Inventory-53

The Brief Symptom Inventory-53 (BSI-53) is a self-reported questionnaire designed to screen the presence of clinical symptoms, specifically Depression (α = 0.892), Anxiety (α = 0.849), Phobic Anxiety (α = 0.795), Somatization (α = 0.876), Interpersonal Sensitivity (α = 0.836), Obsessive-Compulsive traits (α = 0.900), Hostility (α = 0.851), Paranoid Ideation (α = 0.799), and Psychoticism (α = 0.804). The BSI-53, consist on 53 items, each of them scored in a 5-point Likert scale format (Derogatis, 1993), scores closer to 0 indicate a lower symptomatic prevalence, while scores near 4 indicate a higher prevalence. Other authors report good reliability scores for the BSI-53, with an overall Cronbach’s alpha of 0.972 (Mohammad et al., 2019). Previous studies have concluded that the BSI-53 is an objective and precise tool to evaluate the presence of psychopathological symptoms (Ruckenstein and Staab, 2001).

#### Attitudes Toward Seeking Professional Psychological Help Scale–Short Form

The Attitudes Toward Seeking Professional Psychological Help Scale–Short Form (ATSPPH-SF) consists of 10 items with Likert-type responses (Fischer and Farina, 1995). Scores closer to 1 indicate a negative attitude, while scores near 4 indicate a favorable attitude toward seeking professional psychological help (1 = disagree, 2 = somewhat disagree, 3 = somewhat agree, 4 = agree). The scale has also been validated for a Latino adult population, in which an Exploratory Factor Analysis (EFA) suggested the presence of two different dimensions: Openness to Seeking Treatment (α = 0.640; average inter-item *r* = 0.396) and the Perceived Value and Need in Seeking Treatment (VNST) (α = 0.756; average inter-item *r* = 0.526). The overall scale had a Cronbach’s alpha of 0.758 and an average inter-item *r* of 0.461. A Confirmatory Factor Analysis (CFA) based upon the data of the current research validates the two-dimensional nature of the scale proposed by Torres et al. (2020), CFI = 0.972, TLI = 0.963, RMSEA = 0.040.

#### Coronavirus (COVID-19) Awareness Scale

The Coronavirus Awareness Scale-10 (CAS-10) is a 10-item questionnaire built by the authors of the current study, each author individually proposed items, which were later discussed by the research team, the more pertinent and well-structured items were selected to be applied in the selected sample. The CAS-10 has a Likert type response set of 5 points (0–4). An EFA analysis with a maximum likelihood extraction method and an oblimin rotation was executed to detect the underlying factorial structure of the scale. This oblique rotation method allows factors to correlate with each other (Field, 2009), as is the case for many psychological constructs. The Barlett’s Test of Sphericity [χ*^2^* = 1,917.893 (*df* = 45), *p* < 0.001] and the KMO Measure of Sampling Adequacy (0.804) have an acceptable performance (see Table 1). The three resulting factors are Coronavirus Concern (refers to the preoccupation about getting infected with COVID-19), Exaggerated Perception (the belief that the media and governments are overreacting with the COVID-19 situation) and Immunity Perception (the belief that one is not likely to get infected by COVID-19).

**TABLE 1:** Factor loadings for the CAS-10.

	Factor			
Item	Coronavirus concern	Exaggerated perception	Immunity perception	Uniqueness
C4. I am concerned about the spread of Coronavirus (COVID-19).	**0.844**	0.016	–0.008	0.293
C2. I am afraid of catching Coronavirus (COVID-19).	**0.653**	0.063	–0.101	0.549
C10. Coronavirus (COVID-19) is a serious problem.	**0.539**	–0.210	0.090	0.611
C1. I am constantly informed about the situation of the Coronavirus (COVID-19).	**0.431**	–0.009	0.031	0.821
C6. I have taken precautions to avoid getting the Coronavirus (COVID-19).	**0.406**	–0.081	0.063	0.821
C9. I feel like this Coronavirus issue (COVID-19) is more paranoia than anything else.	–.007	**0.861**	–0.003	0.256
C8. I believe that quarantine measures to prevent the spread of Coronavirus (COVID-19) are exaggerated.	–0.058	**0.533**	0.106	0.620
C3. The media exaggerates about the danger of contagion of the Coronavirus (COVID-19).	0.039	**0.492**	0.020	0.765
C7. I am not worried about getting Coronavirus (COVID-19).	–0.011	0.009	**0.989**	0.005
C5. I do not think I can get Coronavirus (COVID-19).	0.068	0.248	**0.301**	0.811

*“Maximum likelihood” extraction method was used in combination with an “oblimin” rotation. RMSEA = 0.053; TLI = 0.943, these values are above the threshold values (RMSEA ≤ 0.08 and TLI ≥ 0.90) used in similar studies (Li et al., 2018). Items C3, C5, C7, C8, and C9 have a negative orientation. “Coronavirus Concern” accounts for 18.204% of the variance, “Exaggerated Perception” for 14.717% and “Immunity Perception” for 11.448%, for a total cumulative percentage of 44.369. Significant item loadings (>0.30) are presented in bold letter.*

Each factor mean is built by averaging the corresponding items raw scores (without reverse coding). Considering item orientation, a higher Coronavirus Concern score (which only contains positive oriented items) indicates a higher Coronavirus Awareness. While high scores on the Exaggerated Perception and Immunity Perception subscales indicate low Coronavirus Awareness. The CAS-10 has a Cronbach’s alpha of 0.762, which is considered acceptable (Coolican, 2004). However, given that this coefficient is affected by the number of items in the scale, average inter-item correlations were also obtained for the overall CAS-10 items (0.436). Specifically, the Coronavirus Concern subscale had the highest Cronbach’s alpha score (α = 0.715; average inter-item *r* = 0.487), followed by the Exaggerated Perception (α = 0.667; average inter-item *r* = 0.436) and Immunity Perception subscales (α = 0.550; average inter-item *r* = 0.381). Given that these average inter-item correlations are between the 0.15 and 0.50 limits, the subscales are considered to be adequately consistent (BrckaLorenz et al., 2013), despite the low number of items included.

### Statistical Analysis

Data was analyzed using Jamovi 1.1 (The Jamovi Project, 2019). First, demographic variables were described using relative and absolute frequencies as well as mean scores and standard deviations. Items from the CAS-10 and the ATSPPH-SF with negative orientation were inversely recoded and their structural properties determined with EFA and CFA, respectively. The relationships between CAS-10, BSI-53, and age were determined by Pearson’s *r* coefficient. Between-group comparisons for sex were made through a MANOVA test, while comparisons for household configuration were determined through a Student’s *t*-test.

Finally, a Mediation Analysis was used to study the relationship between Coronavirus Awareness (predictor), BSI-53 symptoms (mediators) and Attitudes Toward Seeking Professional Psychological Help (outcome). This analysis included Delta method standard errors and bias-corrected percentile bootstrap confidence intervals based on 1,000 replications. Such method provides stable and precise coverage rates and has an overall good performance (Biesanz et al., 2010).

### Ethical Considerations

The current research was made in accordance with the Ethical Guidelines provided by the master’s degree in Clinical Psychology of the National Autonomous University of Honduras, which approved the present study. An online informed consent was presented to all potential participants, it included information regarding the purpose of the study, an anonymity statement, potential risks and benefits, the name and e-mail of the main researcher. At the end of the survey participants were presented with a web link which redirected to a free online psychological assistance website supported by the National Autonomous University of Honduras.

### Coronavirus Awareness and Demographic Variables

#### General Description of Scores

Most respondents are aware of COVID-19 and its implications. For example, 55.7% of the respondents totally agreed with the item “I am constantly informed about the Coronavirus situation” and 69.1% completely agreed that COVID-19 is a serious problem. Nonetheless, a considerable number of participants (30.4%) believed that the media makes exaggerated claims about the COVID-19 dangers (see Table 2). The overall CAS-10 mean score was of 3.007 (*SD* = 0.651), as for the subscales, higher mean scores correspond to Coronavirus Concern (*M* = 3.356, *SD* = 0.641), followed by Exaggerated Perception (*M* = 1.502, *SD* = 1.072) and Immunity Perception (*M* = 1.105, *SD* = 1.048).

**TABLE 2:** Descriptive statistics for each CAS-10 item.

CAS-10 items	Totally disagree n (%)	Disagree n (%)	Neither agree nor disagree n (%)	Agree n (%)	Totally agree n (%)	Mean (SD)
C1. I am constantly informed about the situation of the Coronavirus (COVID-19).	5 (0.60%)	23 (2.7%)	98 (11.5%)	253 (29.6%)	476 (55.7%)	3.371 (0.834)
C2. I am afraid of catching Coronavirus (COVID-19).	64 (7.5%)	70 (8.2%)	133 (15.6%)	190 (22.2%)	398 (46.5%)	2.922 (1.272)
C3. The media exaggerates about the danger of contagion of the Coronavirus (COVID-19).	152 (17.8%)	117 (13.7%)	173 (20.2%)	153 (17.9%)	260 (30.4%)	2.295 (1.469)
C4. I am concerned about the spread of Coronavirus (COVID-19).	15 (1.8%)	12 (1.4%)	79 (9.2%)	194 (22.7%)	555 (64.9%)	3.476 (0.854)
C5. I don’t think I can get Coronavirus (COVID-19).	352 (41.2%)	185 (21.6%)	195 (22.8%)	73 (8.5%)	50 (5.8%)	1.163 (1.218)
C6. I have taken precautions to avoid getting the Coronavirus (COVID-19).	11 (1.3%)	19 (2.2%)	52 (6.1%)	236 (27.6%)	537 (62.8%)	3.484 (0.812)
C7. I’m not worried about getting Coronavirus (COVID-19).	432 (50.5%)	154 (18.0%)	139 (16.3%)	57 (6.7%)	73 (8.5%)	1.047 (1.305)
C8. I believe that quarantine measures to prevent the spread of Coronavirus (COVID-19) are exaggerated.	502 (58.7%)	147 (17.2%)	85 (9.9%)	51 (6.0%)	70 (8.2%)	0.877 (1.284)
C9. I feel like this Coronavirus issue (COVID-19) is more paranoia than anything else.	351 (41.1%)	160 (18.7%)	141 (16.5%)	114 (13.3%)	89 (10.4%)	1.333 (1.392)
C10. Coronavirus (COVID-19) is a serious problem.	12 (1.4%)	13 (1.5%)	77 (9.0%)	162 (18.9%)	591 (69.1%)	3.529 (0.831)

*The table shows raw scores for negative items (C3, C5, C7, C8, and C9).*

#### Coronavirus Awareness and Sex

A MANOVA analysis suggests that there is no statistically significant difference (*p* < 0.05) on the Coronavirus Awareness scores compared to the respondent’s sex. In this sense, the Coronavirus Concern scores for men (*M* = 3.320, *SD* = 0.610) do not differ significantly when compared to female respondents (*M* = 3.377, *SD* = 0.657), *F* (1; 673.939) = –1.244, *p* = 0.205. Male (*M* = 1.596, *SD* = 1.120) and female subjects (*M* = 1.449, *SD* = 1.042) report no significant difference in the Exaggerated Perception scores, *F* (1; 596.423) = 1.928, *p* = 0.059. Similarly, the scores in Immunity Perception do not vary significantly between men (*M* = 1.085, *SD* = 1.025) and women (*M* = 1.116, *SD* = 1.062), *F* (1; 652.178) = –0.417, *p* = 0.674.

#### Coronavirus Awareness and Household Configuration

Respondents who dwelled with children (12 years or younger) had significantly (*p* < 0.05) higher scores in the Exaggerated Perception and the Immunity Perception subscales than people whose households did not have children. No such difference was found in the Coronavirus Concern subscale (*p* = 0.896).

Participants who reported that in their household lived people over the age of 60 had a significantly lower Immunity Perception score than subjects that didn’t have older adults at home (*p* = 0.010). No statistically significant difference was found for the Coronavirus Concern subscale (*p* = 0.162) nor the Exaggerated Perception subscale (*p* = 0.074). Table 3 summarizes the descriptive and comparative statistics for the CAS-10 scale regarding household configuration.

**TABLE 3:** Descriptive and comparative statistics for CAS-10 scores according to household configuration.

Scale	Group	Mean	SD	*F*	df 1; df 2	*p*
**Presence of children at home (<12 years)**						
Coronavirus concern	No	3.353	0.615	0.017	1; 853.00	0.896
	Yes	3.359	0.664			
Exaggerated perception	No	1.422	1.094	4.410	1; 840.615	0.036
	Yes	1.576	1.047			
Immunity perception	No	1.021	1.026	5.121	1; 851.552	0.024
	Yes	1.182	1.064			
**Presence of older adults at home (>60 years)**						
Coronavirus concern	No	3.332	0.643	1.962	1; 697.628	0.162
	Yes	3.395	0.635			
Exaggerated perception	No	1.553	1.079	3.196	1; 701.942	0.074
	Yes	1.419	1.057			
Immunity perception	No	1.176	1.076	6.702	1; 732.372	0.010
	Yes	0.989	0.993			

*From the total sample size, 444 respondents report the presence of minors (<12 years) at home, while 411 do not. On the other hand, 528 report to live with older adults (>60 years) and 327 do not.*

#### Coronavirus Awareness and Age

Results suggest that there is a significant correlation between the respondent’s age and the Coronavirus Awareness subscales. For instance, Coronavirus Concern scores are positively related to the respondent’s age (*r* = 0.116, *p* < 0.001); indicating that increases in Coronavirus Concern scores are weakly associated with increases in age and vice versa. There are also weak negative relationships between the Exaggerated Perception and Immunity Perception subscales regarding the respondent’s age (*r* = –0.171, *p* < 0.001; *r* = –0.097, *p* = 0.004, respectively). It is worth noting that Pearson’s *r* coefficients for all variables are stronger in men than in women (see Table 4).

**TABLE 4:** Relationship between CAS-10 subscales and age, compared by the respondent’s sex.

Scale	Statistics	General age	Men’s age	Women’s age
Coronavirus concern	*r*	0.116***	0.152**	0.096*
	*p*-value	<0.001	0.008	0.024
Exaggerated perception	*r*	−0.171***	−0.225***	−0.136**
	*p*-value	<0.001	<0.001	0.001
Immunity perception	*r*	−0.097**	−0.236***	–0.028
	*p*-value	0.004	<0.01	0.509

Pearson’s r coefficient was used to correlate the variables. *p < 0.05, **p < 0.01, ***p < 0.001.

### Coronavirus Awareness and Mental Health

#### Description of BSI-53 Symptoms

The most intense symptoms reported by the respondents correspond to the Obsessive-Compulsive domain (*M* = 1.595, *SD* = 1.054), followed by Anxiety (*M* = 1.592, *SD* = 0.92) and Interpersonal Sensitivity (*M* = 1.46, *SD* = 1.071). The less intense symptoms included: Hostility (*M* = 1.440, *SD* = 1.021), Social Phobia (*M* = 1.435, *SD* = 1.005), Depression (*M* = 1.378, *SD* = 1.035), Paranoid Ideation (*M* = 1.339, *SD* = 0.937), Psychoticism (*M* = 1.222, *SD* = 0.981), and Somatization (*M* = 1.061, *SD* = 0.897). The Overall BSI-53 score was of 1.391 (*SD* = 0.856).

#### Relationship Between Coronavirus Awareness and BSI-53

Significant (*p* < 0.001), although weak, relationships were found between the Coronavirus Concern subscale and symptoms of Anxiety, Depression, Phobic Anxiety and Psychoticism. Additionally, Exaggerated Perception relates significantly to symptoms of Anxiety, Depression, Hostility, Interpersonal Sensitivity, Obsessive-Compulsive scores, Paranoid Ideation, Psychoticism, and Somatization. Immunity Perception scores are significantly correlated with Paranoid Ideation and Psychoticism. Nonetheless, it’s worth mentioning that all subscale correlation coefficients have a small effect size (*r* < 0.30) (Cohen, 1992). All correlation coefficients between the CAS-10 and the BSI-53 are presented in Table 5.

**TABLE 5:** Correlation between CAS-10 and BSI-53 subscales.

Scale	Statistic	Coronavirus concern	Exaggerated perception	Immunity perception
Anxiety	*r*	0.111**	0.074*	–0.033
	*p*-value	0.001	0.030	0.332
Depression	*r*	−0.088**	0.165***	0.038
	*p*-value	0.01	<0.001	0.269
Phobic anxiety	*r*	0.131***	0.021	–0.06
	*p*-value	<0.001	0.546	0.081
Hostility	*r*	–0.030	0.162***	0.045
	*p*-value	0.383	<0.001	0.186
Interpersonal sensitivity	*r*	–0.060	0.131***	0.049
	*p*-value	0.079	<0.001	0.155
Obsessive-compulsive	*r*	–0.025	0.134***	0.028
	*p*-value	0.463	<0.001	0.413
Paranoid ideation	*r*	–0.008	0.202***	0.077*
	*p*-value	0.820	<0.001	0.024
Psychoticism	*r*	−0.137***	0.201***	0.097**
	*p*-value	<0.001	<0.001	0.004
Somatization	*r*	–0.024	0.13***	0.022
	*p*-value	0.487	<0.001	0.525
Overall BSI-53 score	*r*	–0.019	0.157***	0.034
	*p*-value	0.588	<0.001	0.317

The BSI-53 subscales measure the prevalence of symptoms, but do not constitute a clinical diagnosis. Pearson’s r coefficient was used to correlate the variables. *p < 0.05, **p < 0.01, ***p < 0.001.

#### Coronavirus Awareness and Attitudes Toward Seeking Professional Psychological Help

Coronavirus Concern scores have a significant (*p* = 0.003) and positive (β = 0.178) direct effect on Openness to Seeking Psychological Treatment (OSPT). Moreover, the Exaggerated Perception (β = –0.124, *p* < 0.001) and Immunity Perception scores (β = –0.104, *p* = 0.003) have significant direct negative effects on the VNST scores (see Table 6).

**TABLE 6:** Direct effects of CAS-10 subscales over ATSPPH-SF subscales.

Predictor	Outcome	Estimate	Std. error	*z*-value	*p*	Lower 95% CI	Upper 95% CI
Coronavirus concern	Openness to seeking treatment	0.178	0.060	2.974	**0.003**	**0.039**	0.315
Exaggerated perception		–0.008	0.036	–0.224	0.823	–0.078	0.068
Immunity perception		–0.062	0.036	–1.744	0.081	–0.140	0.012
Coronavirus concern	Value and need in seeking treatment	0.033	0.059	0.569	0.569	–0.097	0.159
Exaggerated perception		–0.124	0.036	–3.451	**<0.001**	–0.205	–0.053
Immunity perception		–0.104	0.035	–2.964	**0.003**	–0.181	–0.029

*Delta method standard errors, bias-corrected percentile bootstrap confidence intervals based on 1,000 replications, ML estimator. Significant estimates (p < 0.05) are in boldface.*

Indirectly, the relationship between Coronavirus Concern and Openness to Seeking Treatment is significantly mediated by Anxiety scores (β = 0.060, *p* = 0.006). Similar results were found for the VNST subscale (β = 0.036, *p* = 0.007). On the other hand, there is a negative significant effect of Immunity Perception over Openness to Seeking Treatment mediated by Paranoid Ideation (β = –0.040, *p* = 0.005) (see Table 7). Overall, Immunity Perception had a negative and significant total indirect effect on Openness to Seeking Treatment (β = –0.029, *p* = 0.005) (see Table 8).

**TABLE 7:** Indirect effects of CAS-10 subscales on ATSPPH-SF subscales.

Predictors	Mediators	Outcomes	Estimate	Std. error	*z*-value	*p*	Lower 95% CI	Upper 95% CI
Coronavirus concern	Anxiety	Openness to seeking treatment	0.060	0.022	2.737	**0.006**	0.022	0.116
	Depression		0.002	0.005	0.451	0.652	–0.005	0.032
	Phobic anxiety		–0.021	0.013	–1.604	0.109	–0.061	0.002
	Hostility		–0.004	0.005	–0.694	0.488	–0.028	0.004
	Interpersonal sensitivity		0.001	0.003	0.375	0.707	–0.005	0.021
	Obsessive–compulsive		0.005	0.009	0.576	0.564	–0.008	0.037
	Paranoid ideation		–0.016	0.010	–1.534	0.125	–0.049	-0.001
	Psychoticism		–0.009	0.009	–0.903	0.366	–0.041	0.003
	Somatization		–0.003	0.006	–0.535	0.593	–0.026	0.008
	Anxiety	Value and need in seeking treatment	0.036	0.013	2.677	**0.007**	0.012	0.072
	Depression		–0.006	0.012	–0.492	0.623	–0.036	0.018
	Phobic anxiety		–0.008	0.006	–1.437	0.151	–0.028	0.001
	Hostility		–0.014	0.011	–1.299	0.194	–0.04	0.007
	Interpersonal sensitivity		–0.006	0.008	–0.739	0.460	–0.027	0.009
	Obsessive–compulsive		0.021	0.011	1.870	0.061	0.002	0.050
	Paranoid ideation		–0.028	0.014	–2.096	**0.036**	–0.060	-0.002
	Psychoticism		0.012	0.012	0.985	0.325	–0.009	0.043
	Somatization		–0.015	0.009	–1.659	0.097	–0.038	-0.001
Exaggerated perception	Anxiety	Openness to seeking treatment	–0.012	0.01	–1.244	0.214	–0.042	0.005
	Depression		0.002	0.004	0.459	0.646	–0.005	0.021
	Phobic anxiety		0.005	0.004	1.108	0.268	–0.001	0.022
	Hostility		0.002	0.003	0.545	0.586	–0.004	0.017
	Interpersonal sensitivity		0.001	0.002	0.253	0.801	–0.005	0.009
	Obsessive–compulsive		–0.004	0.006	–0.742	0.458	–0.023	0.006
	Paranoid ideation		–0.001	0.005	–0.098	0.922	–0.014	0.010
	Psychoticism		0.001	0.003	0.044	0.965	–0.007	0.012
	Somatization		0.004	0.004	0.849	0.396	–0.003	0.018
	Anxiety	Value and need in seeking treatment	0.026	0.017	1.534	0.125	–0.002	0.071
	Depression		–0.002	0.005	–0.478	0.632	–0.029	0.007
	Phobic anxiety		–0.005	0.012	–0.424	0.672	–0.033	0.022
	Hostility		0.001	0.003	0.233	0.815	–0.006	0.017
	Interpersonal sensitivity		0.003	0.008	0.423	0.673	–0.01	0.025
	Obsessive–compulsive		–0.001	0.002	–0.027	0.978	–0.013	0.010
	Paranoid ideation		–0.022	0.013	–1.761	0.078	–0.061	-0.002
	Psychoticism		–0.004	0.009	–0.45	0.653	–0.032	0.009
	Somatization		–0.001	0.002	–0.198	0.843	–0.014	0.006
Immunity perception	Anxiety	Openness to seeking treatment	0.016	0.01	1.523	0.128	–0.002	0.044
	Depression		0.006	0.012	0.529	0.597	–0.019	0.039
	Phobic anxiety		–0.002	0.005	–0.42	0.674	–0.015	0.009
	Hostility		0.002	0.010	0.243	0.808	–0.017	0.025
	Interpersonal sensitivity		–0.015	0.009	–1.724	0.085	–0.043	-0.001
	Obsessive–compulsive		–0.001	0.010	–0.027	0.978	–0.024	0.023
	Paranoid ideation		–0.040	0.014	–2.827	**0.005**	–0.072	-0.013
	Psychoticism		0.005	0.012	0.459	0.646	–0.015	0.032
	Somatization		–0.002	0.008	–0.211	0.833	–0.018	0.016
	Anxiety	Value and need in seeking treatment	–0.005	0.005	–1.032	0.302	–0.028	0.002
	Depression		–0.002	0.004	–0.489	0.625	–0.018	0.005
	Phobic anxiety		0.001	0.003	0.408	0.683	–0.005	0.011
	Hostility		–0.001	0.001	–0.226	0.821	–0.009	0.004
	Interpersonal sensitivity		0.001	0.005	0.266	0.791	–0.009	0.015
	Obsessive–compulsive		0.001	0.002	0.027	0.978	–0.007	0.010
	Paranoid ideation		–0.001	0.007	–0.098	0.922	–0.018	0.013
	Psychoticism		0.001	0.001	0.043	0.965	–0.006	0.010
	Somatization		0.001	0.002	0.206	0.837	–0.005	0.010

*The BSI-53 subscales measure the prevalence of symptoms, but do not constitute a clinical diagnosis. Delta method standard errors, bias-corrected percentile bootstrap confidence intervals based on 1,000 replications, ML estimator. Significant estimates (p < 0.05) are in boldface.*

**TABLE 8:** Total indirect effects of CAS-10 subscales on ATSPPH-SF subscales.

Predictor	Outcome	Estimate	Std. Error	*z*-value	*p*	Lower 95% CI	Upper 95% CI
Coronavirus concern	Openness to seeking treatment	0.015	0.022	0.693	0.488	–0.03	0.06
	Value and need in seeking treatment	–0.009	0.010	–0.831	0.406	–0.035	0.013
Exaggerated perception	Openness to seeking treatment	–0.004	0.008	–0.582	0.561	–0.023	0.015
	Value and need in seeking treatment	–0.004	0.022	–0.184	0.854	–0.056	0.040
Immunity perception	Openness to seeking treatment	–0.029	0.010	–2.813	**0.005**	–0.054	–0.008
	Value and need in seeking treatment	–0.005	0.008	–0.66	0.509	–0.024	0.012

*Delta method standard errors, bias-corrected percentile bootstrap confidence intervals based on 1,000 replications, ML estimator. Significant estimates (p < 0.05) are in boldface.*

Coronavirus Concern had a significant positive total effect on Openness to Seeking Treatment (β = 0.193, *p* < 0.001), while both Exaggerated Perception (β = –0.153, *p* < 0.01) and Immunity Perception (β = –0.109, *p* = 0.002), had significant negative total effects on the VNST scores (see Table 9). However, the overall model only achieved an *r*^2^ of 0.060 for the OSPT and an *r*^2^ of 0.095 for the VNST scores.

**TABLE 9:** Total effects of CAS-10 subscales on ATSPPH-SF subscales.

Predictor	Outcome	Estimate	Std. Error	*z*-value	*p*	Lower 95% CI	Upper 95% CI
Coronavirus concern	Openness to seeking treatment	0.193	0.057	3.366	**<0.001**	0.054	0.325
Exaggerated perception		–0.017	0.036	–0.463	0.643	–0.093	0.054
Immunity perception		–0.067	0.036	–1.849	0.064	–0.145	0.009
Coronavirus concern	Value and need in seeking treatment	0.029	0.056	0.519	0.604	–0.108	0.151
Exaggerated perception		–0.153	0.036	–4.292	**<0.001**	–0.231	–0.079
Immunity perception		–0.109	0.036	–3.072	**0.002**	–0.185	–0.032

*Delta method standard errors, bias-corrected percentile bootstrap confidence intervals based on 1,000 replications, ML estimator. Significant estimates (p < 0.05) are in boldface.*

## Discussion

Our study concluded that individuals who live with older adults (age > 60) had significantly lower Immunity Perception scores than subjects that do not. Complementarily, respondents who live with children (age < 12) tend to endorse the Immunity Perception and the belief that COVID-19 situation is being exaggerated; such beliefs may detonate risky health behaviors in such populations. Results showed that increases in Coronavirus Concern scores are associated with increases in age and vice-versa. This could be related to the fact that older age is considered a risk factor for ARDS and death (Rothan and Byrareddy, 2020). On the other side, younger age has been related to mild disease and better health outcomes (Verity et al., 2020). That is not to say that young people are immune to the virus, therefore, adolescents and young people should receive information about COVID-19. This can be achieved through digital platforms that promote age-friendly content (United Nations Population Fund, 2020).

The most intense symptoms reported by respondents were related to the obsessive-compulsive domain, followed by anxiety and interpersonal sensitivity. These findings are consistent with other studies that reported that individuals exposed to information related to outbreaks experienced higher anxiety symptoms related to health and obsessive-compulsive behavior (Brand et al., 2013). Congruently, our study found a significant positive, although weak, correlation between Coronavirus Concern, anxiety and social phobia. Given the epidemiologic nature of COVID-19, prevention strategies are partly based on social distancing, implying that people should be at least a meter apart from each other (World Health Organization, 2020a). In this sense, many countries around the world recommend their citizens to avoid public spaces (Public Health Ontario, 2020), therefore an increase in social phobia indicators is a natural response to such circumstances.

Another of our findings determined a small negative relationship between the presence of depression symptoms and Coronavirus Awareness. To understand this, the reader must consider that apathy is a common characteristic of depressive disorders (American Psychiatric Association, 2013). In certain populations, respondents with higher depression scores may be less concerned about health issues, indicating a higher level of self-neglect (Hansen et al., 2017). However, these results should be considered with caution as the BSI-53 only screens for symptoms of depression and does not constitute a clinical diagnosis.

Hostility and interpersonal sensitivity symptoms are positively related to the belief that the COVID-19 impact and responses are being exaggerated. Previous research suggests that these traits are highly associated with passive coping strategies (Mao et al., 2003), however, more research is still needed to clearly understand this dynamic. Another symptom of interest corresponds to the paranoid ideation domain, which correlates positively with both the Exaggerated Perception and the Immunity Perception subscales. Respondents with higher paranoid ideation and psychoticism could distrust public media and the government position regarding the impact of COVID-19 in society, minimizing its repercussions. In this sense, prior research has determined a link between paranoid ideation, distrust (Kirk et al., 2013), and the endorsing of conspiracy theories (Darwin et al., 2011).

A similar trend has been suggested for psychoticism and its relationship to medical mistrust and conspiracy theories during pandemic outbreaks (Moukaddam and Shah, 2020), this could help understand the positive relationship between COVID-19 Exaggerated Perception subscale and psychotic symptoms. Although there is a clear relationship between stress exposure and psychotic symptoms (Van Winkel et al., 2008), our research found a negative and weak relationship between Coronavirus Concern and psychoticism. This lack of concern may be explained by the positive correlation of psychoticism with Exaggerated Perception and Immunity Perception. Therefore, respondents who score higher on psychotic symptoms are also less preoccupied with the COVID-19 situation and are more likely to believe that the media exaggerates this situation and that they are not likely to get infected with COVID-19.

This study revealed that most individuals are constantly informing themselves about the COVID-19 current situation and they believe it is a serious problem. However, there is a significant number of respondents that believe the media is exaggerating the situation. Such set of beliefs clearly poses contagion risks that should be addressed by local governments. This could be achieved by promoting health literacy and epidemic prevention strategies (Wang and Wang, 2020), by constantly informing the public about the prevalence and incidence of COVID-19 cases and implementing campaigns designed to educate citizens regarding self-protection measures (Hu et al., 2020).

A relevant finding was that Coronavirus Concern had a positive and direct effect on OSPT, this relationship was significantly mediated by anxiety scores. This is consistent with a study that reported that people who experience anxiety symptoms have, more often, a better attitude toward seeking psychological help (Roness et al., 2005). This could be related to the fact that anxiety symptoms can quickly progress and become harder to manage by oneself in comparison to other disorders. Immunity Perception had a negative effect on OSPT. In this sense, previous research has found that the presence of subjective needs is positively related to attitudes toward seeking psychological help (Nagai, 2015). Translated to our study, this can signify that respondents with high Immunity Perception scores do not feel the need to seek psychological attention. However, given the small determinant coefficients of the results, other variables besides the presence of psychological symptoms should be considered to understand people’s attitudes toward seeking professional psychological help (Nunes Baptista and Zanon, 2017).

Health care systems should take into consideration that the COVID-19 crisis may exacerbate symptoms related to anxiety, depression, and obsessive-compulsive behavior. A way to mitigate this situation could be to provide professional psychological help through the use of online resources, such as they did in China in different stages of the pandemic. In this sense, two simultaneous activities during crisis intervention may be considered: (1) mitigate the fear of the disease and, (2) help coping with difficulties in the adaptation process (Zhang et al., 2020). Since the current pandemic is affecting mental health, the Honduran health care system should take technology as an advantage to reach patients that are experiencing these negative symptoms. Given our results, such response system should focus on interventions designed to mitigate anxiety symptoms. In this sense, recent studies have found that the use of mobile-phone applications based on coach-supported platforms are effective in treating symptoms of anxiety (Graham et al., 2020).

The current research did not took into consideration the potential effects of social distancing and quarantine measures that may impact mental health; more research is yet needed in this subject. Future studies should also focus on specific groups that, given the nature of their work, are in constant risk of infection. Frontline workers (like health care professionals and police force members) have more probabilities of experiencing stressors related to stigmatization, biosecurity procedures, an increased workload and the fear of transmitting COVID-19 to others. Additional variables such as lifestyle and physical activity should also be considered in future studies (Zhou et al., 2020). A mixed-methods approach may provide a more profound comprehension of this phenomenon.

The cross-sectional nature of the current study, which focused on the initial stages of the COVID-19 pandemic, only gives a limited glimpse of the relationship between pandemic outbreaks and mental health indicators of the affected populations. More longitudinal data is still needed on the subject to better determine the causal relationships between variables. Data collection for this research was made between the 16th and the 23rd of March, during this time Honduras reported 30 confirmed COVID-19 cases with 0 fatalities. However, as of May 27th, 2020, the country reported 4,401 confirmed cases and 188 deaths (Honduras Health Secretary, 2020), this increase in COVID-19 cases, in addition to the effects of social confinement and the restriction of liberties, are not accounted for in our research. Another limitation of our study is the non-probabilistic sampling method that was used, which may restrict the inferential capability of our results. Future studies should also explore alternative data analysis, such as non-parametric and Bayesian approaches to further corroborate the results presented in the current research.

The proposed mediation model stated an unidirectional influence of Coronavirus Awareness, mental health indicators and attitudes toward seeking psychological help. However, this relationship could be bidirectional. In this sense, COVID-19 awareness may influence the prevalence of psychopathological indicators. But, such indicators may also have an impact on Coronavirus Awareness itself (as was discussed regarding depression and paranoid ideation symptoms). Further studies are still needed to clarify this dynamic. On the other hand, the BSI-53 measures the prevalence and intensity of different psychological symptoms, but it does not constitute a clinical diagnosis. Finally, given that the current research had a limited psychopathological-based approach to mental health, future studies should include positive and protective variables like resilience, subjective well-being, among other factors that respond to a more holistic, humanistic, and positive concept of mental health.

## Data Availability Statement

The raw data supporting the conclusions of this article will be made available by the authors, without undue reservation.

## Ethics Statement

Ethical review and approval was not required for the study on human participants in accordance with the local legislation and institutional requirements. Written informed consent for participation was not required for this study in accordance with the national legislation and the institutional requirements.

## Author Contributions

ML-B and AL-B were responsible for the questionnaire design, data collection, and statistical analysis. CM-M and CM-S were responsible for building the theoretical and contextual framework of the study, as well as data interpretation. All authors contributed to the article and approved the submitted version.

## Conflict of Interest

The authors declare that the research was conducted in the absence of any commercial or financial relationships that could be construed as a potential conflict of interest.
